# Autologous stem cell transplantation in a patient with scleromyxedema

**DOI:** 10.1002/jha2.463

**Published:** 2022-05-09

**Authors:** Sunita Nathan, Celalettin Ustun

**Affiliations:** ^1^ Division of Hematology Oncology and Cellular Therapy Department of Medicine Rush University Medical Center Chicago Illinois USA

**Keywords:** scleromyxedema, stem cell transplantation

1


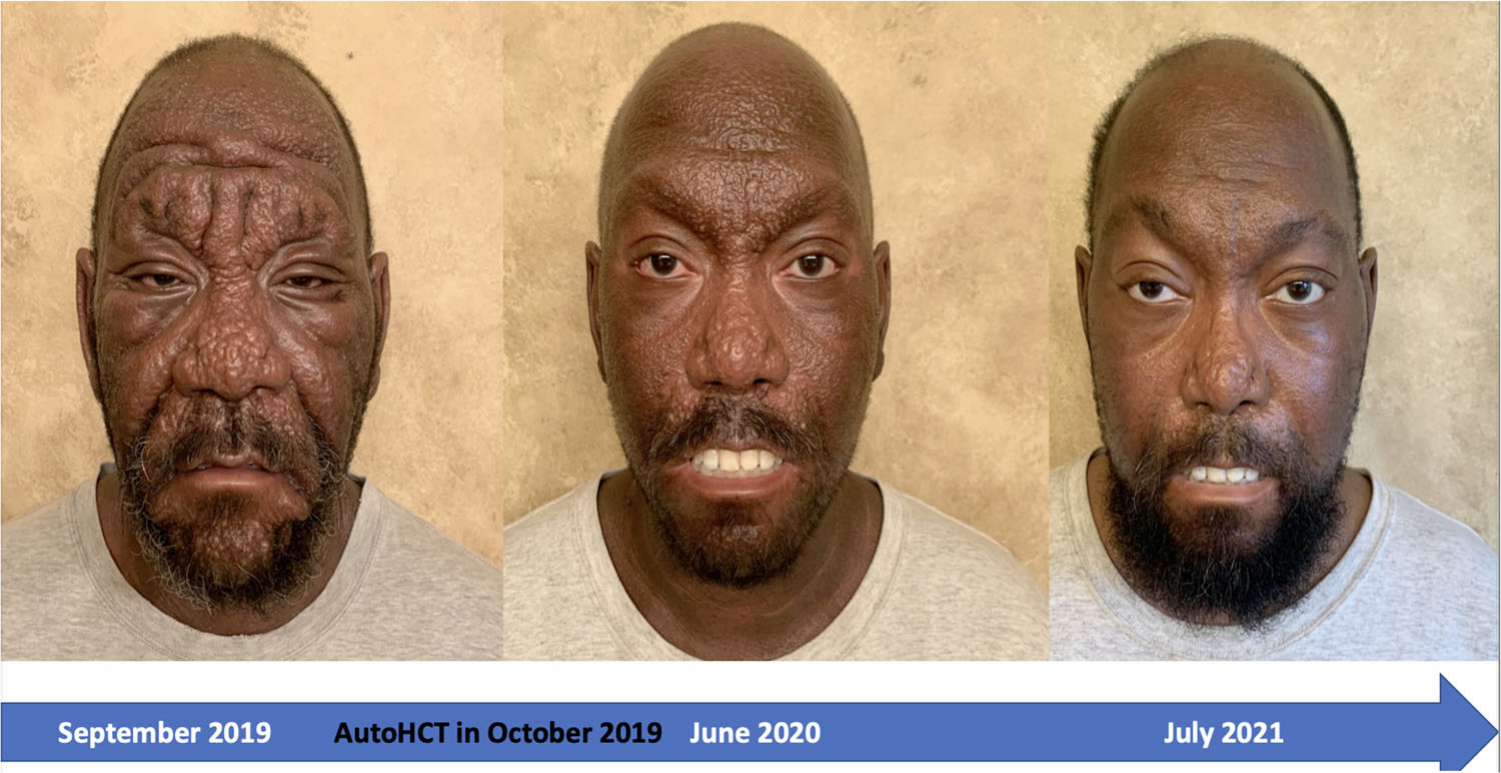


A 46‐year‐old male was diagnosed with scleromyxedema on a skin biopsy in 2014. Scleromyxedema is a rare disorder characterized by papular lichenoid eruption with sclerosing skin infiltration due to the deposition of glycosaminoglycan in the papular and reticular dermis, which leads to thickened skin in almost in entire body. Monoclonal paraproteinemia is a common finding. However, it is associated with disorders of other organs/systems, including gastrointestinal system, cardiopulmonary system, central nervous system, and thus it can be life‐threatening. Because of its similarities with other serious low tumor burden plasma cell diseases (amyloidosis or Polyneuropathy, Organomegaly, Endocrinopathy, Monoclonal protein, Skin changes [POEMS] syndrome), autologous hematopoietic cell transplantation (autoHCT) has been performed with some success in this rare disease with no standard therapy. The mechanism of action of autoHCT is unknown. The patient underwent autoHCT due to failed response to plasma cell‐directed therapies (e.g., lenalidomide, bortezomib) and intravenous immunoglobulin (IVIG). His disease progressed in the skin, also affecting the gastrointestinal system (e.g., not able to open his mouth, esophageal dysfunction) causing malnutrition and thus severe weight loss as well as central nervous system symptoms (e.g., seizure). The picture depicts the patient's face affected by scleromyxedema before and after autoHCT performed in 2019 following high dose melphalan. One can easily see that in addition to skin changes there is improved mouth mobility (he could not open his mouth). Although the patient benefitted from autoHCT in the skin, his clinical course has been complicated by renal and pulmonary complications later in his course requiring high dose steroids and plasmapheresis. The patient recently died of Coronovirus disease 2019 caused by SARS‐CoV‐2 virus (COVID‐19). Scleroderma is a rare, serious systemic disease with unknown etiopathology (e.g., it includes characteristics of plasma cell dyscrasia, autoimmune diseases, or chronic inflammatory diseases). Although autoHCT might be effective in some patients, its treatment remains disappointing.

## CONFLICT OF INTEREST

The authors declare they have no conflicts of interest.

